# Management of Leak by Intraoperative Megastent Insertion During Revisional Bariatric Metabolic Surgery: a Case Report

**DOI:** 10.1007/s11695-023-06943-2

**Published:** 2023-12-07

**Authors:** Mohamed Hany, Mohamed Ibrahim, Mohamed Samir, Ann Samy Shafiq Agayby, Bart Torensma

**Affiliations:** 1 Department of Surgery, Medical Research Institute, Alexandria University, 165 Horreya Avenue, Hadara, Alexandria, 21561 Egypt; 2 Madina Women’s Hospital, Alexandria, Egypt; 3 Leiden University Medical Center (LUMC), Leiden, the Netherlands

**Keywords:** Prophylactic endoscopic stent, Revisional surgery, Sleeve gastrectomy, One-anastomosis gastric bypass

## Abstract

We describe gastric tube continuity restoration (gastrogastrostomy) in a patient who underwent revisional laparoscopic one-anastomosis gastric bypass (OAGB) due to weight recurrence after laparoscopic sleeve gastrectomy (SG). The patient sought restoration to SG due to poor quality of life. A postoperative 11-mm leak at the site of the gastrogastrostomy, attributed to adhesions and edema from a marginal ulcer, complicated the procedure. As a result, laparoscopic exploration was performed, followed by insertion of a megastent. We hereby present video documentation of this case report as well as megastent insertion technique for the treatment of such complications.

**Supplementary Information:**

The online version contains supplementary material available at 10.1007/s11695-023-06943-2.

## Introduction

Endoscopic treatment for leaks after BMS has gained popularity over time [1, 2]. Endoscopic stenting is one of the methods for treating post-bariatric surgery leaks, with a 92% success rate in SG and 96% in RYGB [1]. Stents have downsides such as reflux, chest pain, ulcers, bleeding, nausea, vomiting, and, notably, stent migration (23% rate for all procedures). Pain (6–7%), ulcers (4%), and vomiting (11%) are less frequent issues [3]. A recent meta-analysis of studies on self-expandable metallic stents (SEMS) covering 488 patients revealed an 85.89% (95% CI, 82.52–89.25%) success rate in leak closure. No deaths were directly linked to stent placement, underscoring the method’s safety and effectiveness [1].

## Case Presentation

A 35-year-old male with an OAGB following weight recurrence after an initial SG reported debilitating bowel issues, muscle mass loss, and leg edema presented at the Medical Research Institute, Alexandria University, Egypt. In 2022, he weighed 60 kg (BMI 22 kg/m^2^). He sought an OAGB to SG conversion. The patient and his family declined any hypo-absorptive corrective measures in favor of gastric tube restoration.

The video presented hereby provides a detailed description of the revisional surgical procedure. In summary, during laparoscopy, adhesions and edema were found near the anastomosis site, where the ulcer was adherent to the antrum and colon. The ulcer was torn during dissection, leading to the removal of part of the antrum and anastomosis. Due to tension, reconnecting the gastric pouches was challenging. The antrum was dissected, and the pouches were stapled with an Echelon Flex 60-mm stapler (Ethicon, Cincinnati, OH, USA). The stomach’s edges were well perfused, and hand-sewn closure using V Loc 3.0 (Covidien, MA, USA) over a 40 Fr bougie was performed. Bowel continuity was restored with a side-to-side anastomosis. At the end of the surgery, the air leakage test performed was negative, and intra-abdominal drainage was placed in the vicinity of the gastro-gastrostomy; the total duration of revisional surgery was 140 min with a blood loss of ±200 milliliter. On postoperative day 2, the patient’s high temperature (38.0 °C) and tachycardia (110 beats/min) were observed. After CT scanning, an 11-mm leak was found at the level of the gastro-gastrostomy, and emergency laparoscopic exploration was decided.

### Reoperation

Laparoscopy showed adhesions and exudates managed by suction and lavage. The intestine was tethered, and the pouch was adherent. An intraoperative upper GI endoscopy revealed a fistula. Many options were discussed in the team, including (1) drainage and jejunostomy feeding (challenged by some as the intestine was tethered and the proximal jejunum was inaccessible), (2) drainage and nasoduodenal tube insertion (an accepted option for some), (3) turning the leak into a controlled fistula by inserting a T-tube through the rent, with drainage and nasoduodenal feeding or internal pigtail drainage (IPD) and nasoduodenal feeding (challenged by some due to the uncertainty of the T-tube being held in place, the fact that the rent was not draining into a contained cavity so IPD maybe not a suitable choice, the high possibility of stricture formation with healing, and lack of experience with the T-tube), and (4) insertion of a metal stent with the advantages of immediate isolation of the fistula, early resumption of enteral feeding without tubes, and the satisfactory experience of the team with the use of megastents for leaks after SG. Eventually, a 23-cm Niti-S Mega esophageal stent (Taewoong Medical, South Korea) was placed post-discussion. The stent was tested and confirmed with water and airtight tests. New intra-abdominal drains were inserted (pelvis and surgical bed) (Stent Insertion Procedure Appendix 1). The total duration of reoperation was 65 min with a blood loss of ±50 ml.

###  Postoperative Course After Relaparoscopy

The patient received total parenteral nutrition for a week. On postoperative day 2, the patient began oral fluids for 3 days, transitioned to pureed food for a week, and then gradually ate as tolerated with a protein isolate supplement. Both drains were removed the day before discharge, and the patient was discharged on postoperative day 15. A follow-up CT scan revealed reduced air at the gastric leak site, as well as presence of bilateral pleural effusion treated medically. The stent was checked weekly by abdominal X-ray and removed after 5 weeks. A CT scan the next day showed no contrast leakage, and the patient’s subsequent recovery was uneventful.

## Conclusion

Revisional surgeries should be conducted in facilities that possess adequate equipment and a competent staff. In cases where patients experience an acute leak measuring more than 1 cm, an intraoperative fully covered SEMS can be employed. This technique effectively manages the leak and facilitates early oral intake, particularly when alternative methods are unsuitable due to friable tissue or extensive peritonitis. Nevertheless, it is vital to closely monitor the patient to prevent any complications related to the stent.

### Supplementary Information

ESM 1 (MP4 184876 kb)
